# Natural Flt3Lg-Based Chimeric Antigen Receptor (Flt3-CAR) T Cells Successfully Target Flt3 on AML Cell Lines

**DOI:** 10.3390/vaccines9111238

**Published:** 2021-10-25

**Authors:** Varvara Maiorova, Murad D. Mollaev, Polina Vikhreva, Elena Kulakovskaya, Dmitry Pershin, Dmitriy M. Chudakov, Alexey Kibardin, Michael A. Maschan, Sergey Larin

**Affiliations:** 1 Dmitry Rogachev National Medical Research Center of Pediatric Hematology, Oncology and Immunology, 117997 Moscow, Russia; murad.mollaev@fccho-moscow.ru (M.D.M.); polina266@gmail.com (P.V.); elena.kulakovskaya@fccho-moscow.ru (E.K.); dmitry.pershin@fccho-moscow.ru (D.P.); alexey.kibardin@fccho-moscow.ru (A.K.); Michael.Maschan@fccho-moscow.ru (M.A.M.); sergey.larin@fccho-moscow.ru (S.L.); 2 Center of Life Sciences, Skolkovo Institute of Science and Technology, 121205 Moscow, Russia; Chudakovdm@gmail.com; 3 Institute of Translational Medicine, Pirogov Russian National Research Medical University, 117997 Moscow, Russia; 4 Shemyakin-Ovchinnikov Institute of Bioorganic Chemistry, 117997 Moscow, Russia

**Keywords:** ligand-based targeting, CAR T cell therapy, acute myeloid leukemia

## Abstract

Relapsed/refractory acute myeloid leukemia (AML) cannot be cured with chemotherapy alone, as the blasts survive the treatment. Chimeric antigen receptor (CAR) approaches for AML are being actively developed. CARs promote immune reactions through recognition of the target molecular epitopes at the surface of cancer cells. The recognition involves the extracellular portion of the CAR protein, which corresponds to either the antibody or the physiological binding partner of the targeted antigen. Here, we design a chimeric receptor with a full-length natural Flt3-ligand recognition module that targets Flt3 tyrosine kinase, known as an adverse marker in AML. We demonstrate specific killing of Flt3-positive THP-1 cells by Flt3-CAR T cells and the lack of cytotoxicity towards Flt3-negative U937 cells. We prove that the inherent cytolytic capacity of T cells is essential for the killing. Finally, we confirm the authenticity of targeting by its competitive dose-dependent inhibition with a soluble Flt3-ligand. The developed system can be viewed as a non-immunogenic functional equivalent of scFv-mediated targeting. The robust in vitro antitumor effects of Flt3-CAR T cells, combined with their low off-target cytotoxicity, hold promise for AML treatment.

## 1. Introduction

Acute myeloid leukemia (AML) is a hematopoietic neoplasm defined by misregulated clonal expansion of myeloid progenitor cells (blasts). First-line treatment for AML is based on intensive chemotherapy on the backbone of cytarabine and anthracycline. A significant proportion of patients require hematopoietic stem cell transplantation (HSCT) to secure long-term survival [1]. Both frontline chemotherapy and HSCT have been associated with multiple adverse effects, including long-term sequelae. Despite the intensity of the treatment approaches, leukemic blasts may develop resistance. The clear impossibility of the further intensification of the standard chemotherapy regimens necessitates the development of alternative and complementary treatment modalities, including, above all, immunotherapy.

Early steps in cancer immunotherapy involved the creation of vaccines, e.g., peptide vaccines, whole-cell vaccines, and dendritic cell-based vaccines, aimed at stimulating anticancer immune responses. For example, the primed dendritic cells were expected to promote the maturation of cytotoxic lymphocytes armed against tumor cells. This concept, on its own, failed to provide effective antitumor therapy [2]. Yet, the attempts to treat tumors with vaccines immensely advanced our understanding of immune system functionalities and fine regulation in humans [3]. Cancer vaccines showed significantly lower systemic toxicity than chemotherapy, which inspired further research [4,5] in several important directions, notably the chimeric antigen receptor (CAR) T cell therapy.

The development of CAR T cell therapies for AML is currently a mainstream. Autologous T cells are modified with engineered genetic constructs delivered by a variety of methods. The cells start to express CARs as integral membrane proteins. Extracellular portions of these proteins recognize a tumor-specific target antigen expressed on blast cells. Upon recognition, T cells become activated and destroy the blasts. Such CAR-mediated cytotoxicity is independent of co-stimulatory signals from conventional antigen-presenting cells and results in the specific elimination of all cells which expose the target at their surface. Endowed with CAR expression constructs, the autologous patient T cells become specifically trained to attack the pathological cells of the tumor.

The therapeutic output of CAR-mediated cytotoxicity depends on two conditions: target choice and binding affinity. Several candidate antigens are being explored as potential targets in AML [6]. Unfortunately, most of them are expressed by normal hematopoietic progenitor cells, with the consequent risks of pronounced on-target off-tumor cytotoxicity [7,8,9]. The situation with affinity is more encouraging. Classical CAR designs employ antigen-binding domains derived from specific immunoglobulin molecules. An alternative approach is to target a particular surface receptor by mimicking its physiological (non-immunoglobulin) binding partner. In this article, we describe the development of CAR with a natural ligand moiety, targeting tyrosine kinase Flt3 (Fms-like tyrosine kinase 3, also known as CD135).

Tyrosine kinase growth factor receptor Flt3 is expressed on the surface of early hematopoietic precursors and mature dendritic cells. The Flt3-mediated signaling drives cell survival and proliferation; its natural ligand Flt3Lg initiates homodimerization and activation of the receptor complex. Abnormal activation of Flt3 is highly typical for AML. Approximately 25% of adult and 5-10% of pediatric AML patients harbor Flt3 internal tandem duplication (Flt3-ITD), which has been associated with inferior survival. The Flt3-ITD mutation provides constitutive anti-apoptotic and proliferation signaling. In about 75% of the patients with Flt3-ITD, the mutation persists at relapses. Moreover, up to 16.7% of AML patients with unaltered (wild-type) Flt3 at diagnosis subsequently acquire ITD mutation. All in all, Flt3 represents a plausible therapeutic target in AML.

Recently, several groups have been working on Flt3-CAR T cells for AML therapy. Their effectiveness against Flt3- and Flt3-ITD-positive cells in vitro and in vivo was shown [10,11]. According to [11], crenolanib stimulated Flt3-ITD exposure on the cell surface, thus increasing targeting efficiency. The Flt3-CAR T Amg553 variant is currently participating in a Phase I clinical study [12]. Flt3-CAR T was also shown to target Flt3-positive HSCs [13,14]. Although, despite active research, up to date Flt3-CAR T cells were not described to be cytotoxic against mature dendritic cells present throughout of the body, even though the cross-reactivity of human and mouse Flt3 and Flt3Lg is well-known [15].

Mouse, humanized or human antibody-derived single-chain variable fragments (scFv) are commonly used in CAR design [16]. Although humanized scFv are less immunogenic than mouse alternatives, possible non-specific interactions cannot be neglected. Certain scFv may support the off-target CAR T cell activation. For this study, we chose to target the antigen with its natural ligand, Flt3Lg, thus minimizing the risks of both immunogenicity and off-target interactions. The target recognition module of our Flt3-CAR comprises full-length sequences of human Flt3Lg (soluble isoform) instead of scFv.

We produce Flt3-CAR T cells and demonstrate the high specificity of their activation. Using an in vitro AML model, we show that the engineered Flt3-CAR T cells promote robust elimination of Flt3-positive blasts while showing zero cytotoxicity towards Flt3-negative blasts. Finally, we prove that Flt3-CAR interacts with Flt3 by its Flt3Lg module, thus confirming the authenticity of the observed effects.

## 2. Materials and Methods

### 2.1. Generation of CAR and mKate2 Lentiviruses

The Met1-Pro185 region of Flt3Lg cDNA was amplified by RT-PCR and cloned in frame with CD8hinge-ICOS-4-1BB-CD3ζ-P2A-EYFP (EYFP stands for enhanced yellow fluorescent protein coding fragment) or CD8hinge-ICOS-4-1BB-CD3ζ into pLVX-EF-1α (Takara, Kusatsu shi, Japan). Anti-CD19-CAR was generated by cloning scFv FMC63 in frame with CD8hinge-ICOS-4-1BB-CD3ζ into pLVX-EF-1α (Takara). 

The pmKate2 vector (Evrogen, [17]) was used as a template to introduce human c-Myc nuclear localization signal (NLS) at the C-terminal end of mKate2 by PCR. The product was subcloned into pLVX-EF-1α (Takara) vector.

The lentiviruses were packaged using HEK293T cell line. HEK293T cells were transfected with Lenti-X Packaging Single Shots (VSV-G) (Takara) according to optimized commercial protocol. The virus-containing supernatant was collected 48 h post-transfection, concentrated using Lenti-X Concentrator (Takara), aliquoted, and preserved at −80 °C until use.

### 2.2. Generation of CAR T Cells, CAR Jurkat Cells, and THP-1-mKate2 and U937-mKate2 Cell Lines

All cultures were maintained at 37 °C in a humidified atmosphere of 5% CO_2_. Primary donor T cells were isolated by apheresis on the CliniMACS Prodigy^®^ (Miltenyi Biotec). T cells were seeded in 24-well plates at 1 × 10^6^ cells per well, activated with TransAct reagent (Miltenyi Biotec, Bergisch Gladbach, Germany), transduced with CAR gene cargo viruses, and expanded for 7 days in TexMacs medium (Miltenyi Biotec). The culture medium was supplemented with IL-7 and IL-15 (25 ng/mL, Miltenyi Biotec). Jurkat cells were seeded in 24-well plates at 5 × 10^5^ cells per well and transduced with CAR gene cargo viruses. Transduction efficiency was estimated by flow cytometry. EYFP-synthesizing cells were regarded as CAR-positive.

THP-1 and U937 cells were seeded at 5 × 10^5^ cells per well and transduced with mKate2 cargo viruses. After transduction, the THP-1-mKate2 and U937-mKate2 cells were subjected to puromycin selection to eliminate non-transduced cells. Selection efficiency was confirmed by flow cytometry.

### 2.3. Western Blotting

The transduced CAR T cells and CAR Jurkat cells were collected, washed with Dulbecco’s Phosphate Buffered Saline (DPBS, HyClone), and lysed in Laemmli sample buffer supplemented with β-mercaptoethanol (Bio-Rad, Hercules, CA, USA). The samples were boiled for 10 min at 100 °C before loading. The proteins were run on SDS-polyacrylamide gel and transferred onto polyvinylidene difluoride (PVDF) membrane (GE Healthcare, Chicago, IL, USA). The membrane was blocked overnight in 1% ECL Block (GE Healthcare). CARs were stained with anti-CD3ζ-chain antibody (Sigma) detected with anti-Rabbit-HRP (GE Healthcare). EYFP was stained with anti-GFP antibody (SCI-Store) detected with anti-Mouse-IgG-HRP (GE Healthcare). 

### 2.4. Cell Culture

Human HEK293T, Jurkat, THP-1, and U937 cells were obtained from the Russian Cell Culture Collection of Vertebrate Cells (Institute of Cytology, Russian Academy of Sciences, St. Petersburg, Russia).

All cell lines were maintained at 37 °C in a humidified atmosphere of 5% CO_2_. THP-1, THP-1-mKate2, U937, and U937-mKate2 cells were cultured in complete RPMI medium (Gibco) with 10% FBS (Gibco) and 2 mM L-Gln. Transduced Jurkat cells were cultured similarly, with the addition of 100 U/mL penicillin and 100 µg/mL streptomycin. HEK293T cells were cultured in complete DMEM medium (Gibco) with 10% FBS (Gibco), 2 mM L-Gln, 100 U/mL penicillin, and 100 µg/mL streptomycin.

### 2.5. Flow Cytometry

THP-1 and U937 cells were washed with PBS, stained with anti-CD135-PE conjugated antibodies (Beckman Coulter, Brea, CA, USA) for 15 min, washed again, and counted on a Navios flow cytometer (Beckman Coulter).

### 2.6. CAR T and CAR Jurkat Cell Cytotoxicity Assay

Transduced T cells were rested under cytokine starvation for 3 days and transferred to RPMI complete medium. The 96-well plates for the assay were pre-coated with 10 µg/mL human plasma fibronectin (IMTEK) and washed with DPBS. CAR T cells were mixed with THP-1-mKate2 or U937-mKate2 cells at an effector-to-target ratio (E:T) of 1:1 or 5:1. The cells were incubated for 4 days (37 °C, 5% CO_2_) in the IncuCyte^®^ S3 Live-Cell Imaging System (Sartorius, Göttingen, Germany). Each well was imaged at 9 locations; all experiments were repeated at least 3 times.

For CAR Jurkat cell-mediated killing tests, 96-well plates were prepared similarly. CAR Jurkat and THP-1-mKate2 cell suspensions were mixed at E:T = 1:1. The cells were incubated for 3 days (37 °C, 5% CO_2_) and analyzed similarly.

The competitive Flt3Lg binding experiments involved soluble Flt3Lg (SCI-Store) in final concentrations of 100, 20, 2, and 0.2 ng/mL. The ligand was added to the coculture medium before mixing of cell suspensions.

### 2.7. Cell Proliferation Assay

Proliferation of THP-1 cells in the presence of Flt3Lg was measured by optimized MTS assay. The cells were washed with serum-free RPMI medium and seeded in a 96-well plate, in low-serum RPMI medium. Soluble Flt3Lg (SCI-Store) was titrated from 30 ng/mL by 3-fold serial dilutions. Proliferation was assessed using CellTiter 96^®^ AQueous One Solution Cell Proliferation Assay kit (Promega, Madison, WI, USA) according to the manufacturer protocol.

### 2.8. Quantitative Data Handling

Statistical analysis of flow cytometry data was performed with Kaluza software. For the MTS assay, all measurements were made in triplicates and represented as the mean values. All experiments were repeated at least 3 times.

## 3. Results

### 3.1. Generating Flt3-CAR 

To target Flt3, we engineered a chimeric antigen receptor Flt3-CAR comprising full-length polypeptide sequence of Flt3Lg (soluble isoform) known to bind Flt3 with high affinity (2.5 × 10^10^ M^−1^ [18]). The Flt3Lg moiety in the extracellular part of Flt3-CAR is supposed to be non-immunogenic, as it corresponds to a real human cytokine.

**Figure 1:**
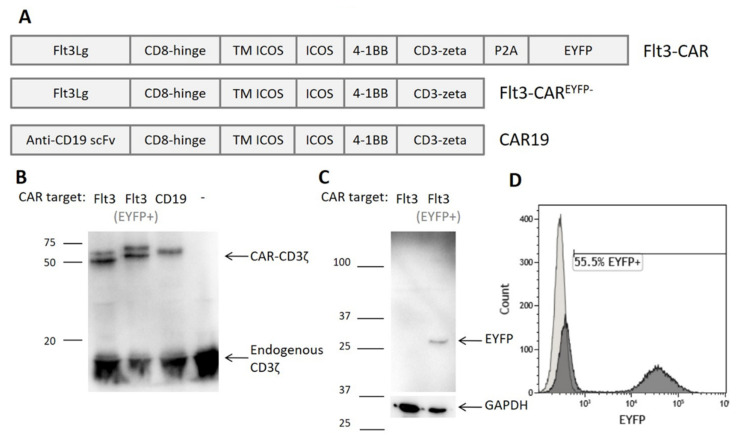
Generation of Flt3-CAR T cells. (**A**) Domain structure of Flt3-CAR, Flt3-CAR^EYFP-^, and CAR19 coding regions (EYFP stands for enhanced yellow fluorescent protein). (**B**) Western blot demonstrating synthesis of chimeric receptor proteins in transduced cells. High bands corresponding to CD3ζ domain of the chimeric receptors (50–60 kDa) are observed in all lanes except negative control (non-transduced T cells). Lower bands corresponding to the CD3ζ part of the endogenous T cell receptor complex (16 kDa) are present in all lanes. (**C**) Western blot demonstrating synthesis and proper cleavage of EYFP protein in Flt3-CAR T cells. The arrow indicates a single band of appropriate size for EYFP (27 kDa). (**D**) A representative flow cytometry histogram showing EYFP synthesis in Flt3-CAR T cells on day 7 after transduction.

Our Flt3-CAR contains a combined ICOS and 4-1BB co-stimulatory domain and a classical CD3ζ activating domain. It is a natural ligand-based third generation CAR intended for targeting Flt3 receptors on blast cells. The EYFP tag, attached to the C terminus via a self-cleaving P2A peptide linker, facilitates the evaluation of transduction efficiency. We also produced gene constructs encoding Flt3-CAR without EYFP (Flt3-CAR^EYFP-^) and CAR19 (targeting CD19 antigen; Figure 1A).

Primary donor T cells were activated, transduced with CAR gene cargo lentiviruses, and analyzed by Western blotting. High bands corresponding to CD3ζ domain of CAR were observed for all studied constructs (Figure 1B). CAR19 T cells were used as a positive control. For both Flt3-CAR and Flt3-CAR^EYFP-^, bands of the same weight were successfully detected. The double bands observed for Flt3-CARs apparently reflect partial glycosylation of the Flt3Lg moiety. The same cell lysates were used for anti-EYFP immunoblotting, demonstrating the proper cleavage of EYFP and the absence of residual fusions (Figure 1C).

Based on these findings, EYFP-synthesizing cells were regarded as CAR-positive. Relative counts of EYFP-positive cells indicated the average transduction efficiency of 49% (Figure 1D).

### 3.2. Flt3-CAR T Cells Specifically Kill Flt3-Positive Cells

**Figure 2:**
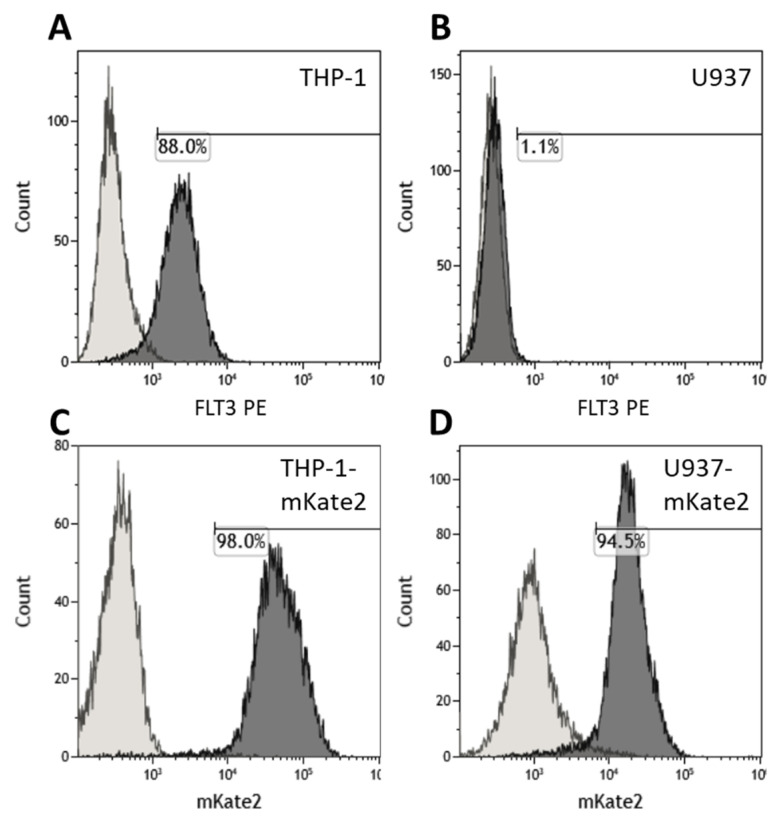
Characterization of the target cells lines THP-1, U937, THP-1-mKate2, and U937-mKate2 by flow cytometry. (**A**,**B**) Levels of positivity for Flt3 in THP-1 and U937 populations, respectively, measured by staining with anti-CD135-PE antibody. (**C**,**D**) The 98.0% positive THP-1-mKate2 and 94.5% positive U937-mKate2 were obtained by transduction of THP-1 and U937 cell lines with mKate2 followed by selection on puromycin.

Human cell lines THP-1 (Flt3+) and U937 (Flt3-) were used as AML model. The immunophenotypes [19] were confirmed by staining with anti-Flt3-PE antibodies (Figure 2A,B). Both target cell lines were transduced with mKate2 cargo lentivirus to make them fluorescent (Figure 2C,D) and thereby distinguishable from the CAR T cells during co-incubation experiments.

**Figure 3:**
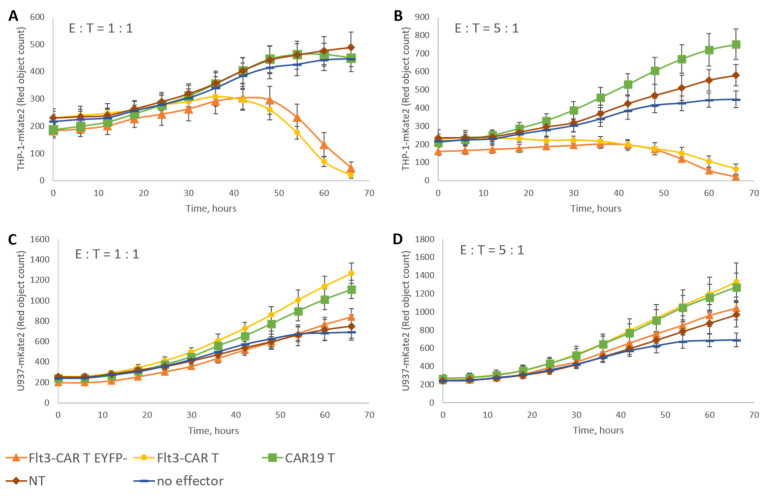
Specific Flt3-CAR T cell-mediated killing of target cells in vitro. Flt3-CAR T cells, Flt3-CAREYFP-T cells, CAR19 T cells, and non-transduced (NT) cells were co-incubated with THP-1-mKate2 cells at E:T = 1:1 (**A**) or 5:1 (**B**), or U937-mKate2 cells at E:T = 1:1 (**C**) or 5:1 (**D**). A color/shape legend for the lines and data points is given in the figure. ‘Red object counts’ acquired by IncuCyte^®^ reflect the viability of target (THP-1-mKate2 or U937-mKate2) cells. The data are represented as means ± SD.

The rested Flt3-CAR T cells, Flt3-CAR^EYFP-^ T cells, CAR19 T cells, and non-transduced (NT) T cells were co-incubated with THP-1-mKate2 or U937-mKate2 for 4 days; the initial E:T ratio was 1:1 or 5:1. Flt3-CAR T cells and Flt3-CAR^EYFP-^ T cells showed specific cytotoxicity towards Flt3-positive THP-1-mKate2 in all experiments (Figure 3A). Both control CAR19 T cells and non-transduced T cells showed no cytotoxicity towards THP-1-mKate2, even at E:T = 5:1 (Figure 3B). Flt3-CAR T cells and Flt3-CAR^EYFP-^ T cells showed similar cytotoxic activity. None of the CAR T cells generated in this study interfered with the growth of Flt3-negative U937-mKate2 cells (Figure 3C,D).

### 3.3. Specific Cytolytic Capacity of T Cells Is Necessary for Flt3-CAR-Mediated Killing

**Figure 4:**
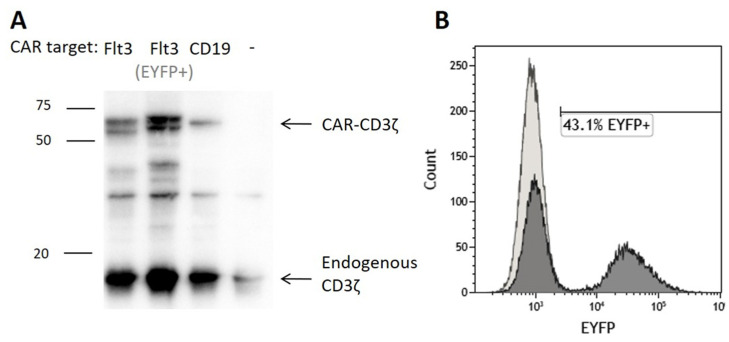
Generation of Flt3-CAR Jurkat cells. (**A**) Western blot demonstrating synthesis of the chimeric receptor proteins in transduced cells. High bands corresponding to CD3ζ domain of the chimeric receptors (50–60 kDa) are observed for transduced Jurkat cells and not observed for non-transduced Jurkat cells. Lower bands corresponding to CD3ζ part of the endogenous T cell receptor complex (16 kDa) are observed in all lanes. (**B**) A representative flow cytometry histogram showing EYFP synthesis in Flt3-CAR Jurkat cells on day 7 after transduction.

To assess the significance of T cell functional identity for Flt3-CAR-induced killing, we developed a Flt3-CAR T cell model with impaired cytolytic activity. We used Jurkat cells as a non-cytotoxic T cell equivalent and transduced them with Flt3-CAR, Flt3-CAR^EYFP-^ and CAR19 gene cargo lentiviruses (Figure 1A). Upon transduction, Jurkat cells produced Flt3-CAR, Flt3-CAR^EYFP-^, and CAR19 identically with the donor-derived T cells, as confirmed by Western blotting (Figure 4A). Similar bands of appropriate size were detected with anti-CD3ζ staining. Transduced with Flt3-CAR, the Jurkat population was 43.1% EYFP-positive (Figure 4B). Eventually, we developed Jurkat cell lines expressing Flt3-CAR, Flt3-CAR^EYFP-^ and CAR19 proteins.

**Figure 5:**
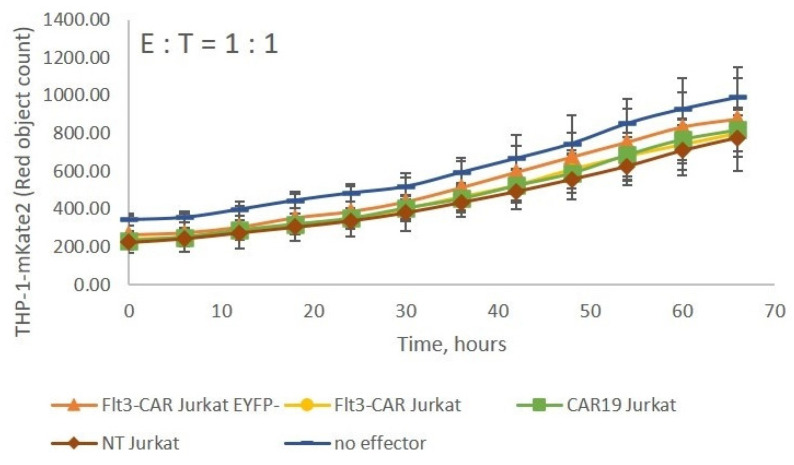
Flt3-CAR Jurkat cells lack cytotoxicity towards THP-1-mKate2 cells. Flt3-CAR Jurkat cells, Flt3-CAREYFP- Jurkat cells, CAR19 Jurkat cells, and non-transduced (NT) Jurkat cells were co-incubated with THP-1-mKate2 cells at E:T = 1:1. A color/shape legend for the lines and data points is given in the figure. ‘Red object counts’ acquired by IncuCyte^®^ reflect the viability of THP-1-mKate2 cells. The data are represented as means ± SD.

The Flt3-CAR Jurkat, Flt3-CAR^EYFP-^ Jurkat, CAR19 Jurkat, and non-transduced Jurkat cells were co-incubated with Flt3-positive THP-1-mKate2 cells at E:T = 1:1. None of the effectors interfered with the growth of the target cell line (Figure 5). The results indicate that unimpaired T cell functionality is a prerequisite for Flt3-CAR-induced killing.

### 3.4. Soluble Flt3Lg Promotes Proliferation of THP-1 Cells

**Figure 6:**
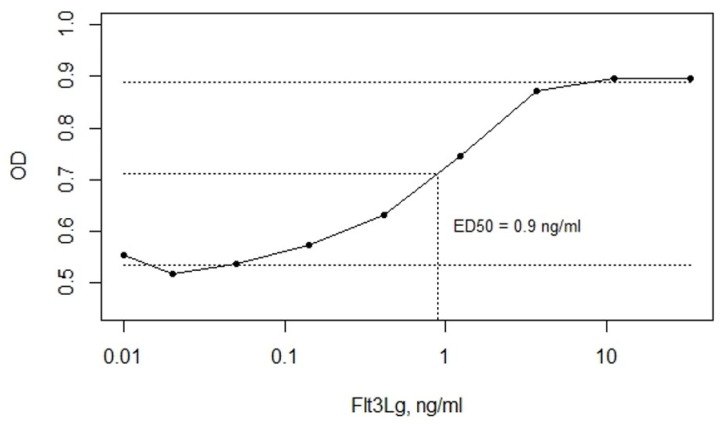
The soluble Flt3Lg-induced proliferation of THP-1 cells, evaluated by MTS assay, with ED50 = 0.9 ng/mL determined using mean OD values of lower and upper plateaus. The data are represented as means ± SD. OD stands for sample optical density.

Flt3 is known to mediate the abnormal proliferation of AML blasts [20]. The acute monocytic leukemia-derived THP-1 cells express wild-type Flt3. Using an optimized MTS assay, we showed that soluble Flt3Lg stimulates THP-1 proliferation (Figure 6). With the addition of recombinant human Flt3Lg, the proliferation of THP-1 cells increased in a dose-dependent manner (ED_50_ = 0.9 ng/mL) reaching saturation at 10 ng/mL.

### 3.5. Flt3-CAR T Cells Recognize the Flt3Lg-Binding Site of Flt3 on the Surface of THP-1 Cells

**Figure 7:**
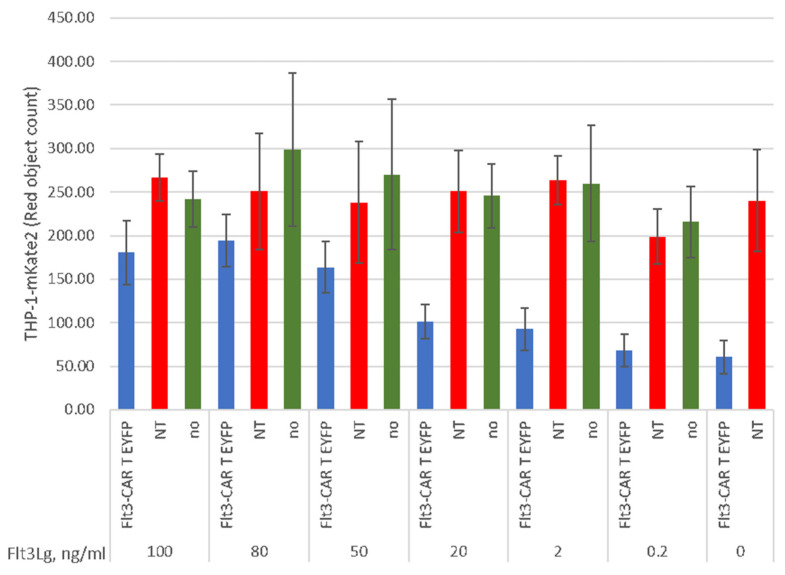
Dose-dependent inhibition of Flt3-CAR T cell-mediated killing with soluble Flt3Lg. The ligand was added at indicated concentrations to the co-incubation medium prior to the experiment. THP-1-mKate2 cells were co-incubated with Flt3-CAR T cells (blue bars), non-transduced T cells (red bars), or no T cells (green bars). ‘Red object counts’ acquired by IncuCyte^®^ over 45 h co-incubation reflect the viability of THP-1-mKate2 cells. The data are represented as means ± SD.

The recognizing part of Flt3-CAR comprises wild-type Flt3Lg, which is presumed to interact with Flt3 via the Flt3Lg-binding surface. To test the authenticity of such recognition, we performed a series of functional tests in the presence of soluble Flt3Lg. The soluble human Flt3Lg was added to culture medium upon co-incubation of THP-1-mKate2 cells with Flt3-CAR T cells, non-transduced T cells, or no effector cells for 20 h. The addition of soluble Flt3Lg led to the dose-dependent inhibition of Flt3-CAR T-mediated killing of THP-1 cells (Figure 7). An excess of soluble Flt3Lg (100 ng/mL, which exceeded the saturating concentration 10-fold), markedly interfered with the cytotoxic effects, while lower concentrations of Flt3Lg (2 ng/mL and 0.2 ng/mL, around ED_50_ doses) did not prevent the Flt3-CAR T cell activity. These experiments confirm that the Flt3-CAR receptor recognizes its target molecule, Flt3, via the Flt3Lg-binding site of the receptor.

## 4. Discussion

The main obstacle in developing CAR T cell therapy for AML is the lack of a suitable antigen for specific blast cell recognition. Most of the candidate target molecules for AML are also expressed on hematopoietic progenitor cells [21]. Among the characterized tumor-associated antigens, growth factor receptors are of particular interest. The dysregulation of normal paracrine signaling by aberrant activity of growth factor receptors is typical in cancers [22,23]. Often, pathological cells (blasts) can be discriminated from non-leukemic cells by elevated expression of non-mutated growth factor receptors.

Two major strategies for the alleviation of the abnormal growth factor receptor signaling are inhibition of the excessive signaling [24] and the targeted elimination of the transformed cells [21]. Several molecular targets for CAR T cell-mediated elimination of AML blasts have been considered. However, the killing of tumor cells is often accompanied by the elimination of normal hematopoietic cells, which also carry target antigens on their surfaces.

A number of projects have focused on the design and selection of single-chain antibody fragments to be used in chimeric receptors [25,26,27]. The efficiency of CD123-CAR T cell-mediated killing was shown to correlate with the density of the antigen molecules on the target cells [26]. This finding inspired a massive screening for optimal affinity among slightly different scFv, ultimately yielding CAR T cells capable of distinguishing AML blasts from normal cells. Another group [27] introduced point mutations into anti-CD123 scFv to modulate its affinity, thereby reducing the cytotoxicity towards normal cells. In this study, we used a full-length natural ligand (Flt3Lg) of the target molecule (Flt3) as a recognition module in chimeric receptor Flt3-CAR. The estimated affinity of binding between Flt3 and Flt3Lg (2.5 × 10^10^ M^−1^ [18]) is high enough to ensure effective targeting.

A similar approach was reported by Wang et al. [15], who used the isolated receptor-binding domain of Flt3Lg as a recognition module in chimeric receptors. The developed FLT3L CAR T cells showed specific activity towards Flt3-positive THP-1, MV4-11, and MOLM13 cell lines, albeit that they also showed a weak cytotoxic effect towards the Flt3-negative cell line U937 as soon as effector : target ratio (E:T) = 1:1. The full-length sequence of soluble human Flt3Lg cytokine used by us in this study is about 50 amino acids longer than the fragment used by Wang et al. [15]. We demonstrate that Flt3-CAR with the full-length soluble Flt3Lg polypeptide does not affect the proliferation of the Flt3-negative U937 cells, even at E:T = 5:1, indicating negligible off-target activity and higher specificity of activation compared with the use of an isolated binding domain [15]. Moreover, we show that an excess of soluble Flt3Lg effectively inhibits the interaction of Flt3-CAR with Flt3. Based on this result, we conclude that Flt3-CAR recognizes its target directly through the Flt3Lg binding site.

The non-specific activation of chimeric receptors can be pronounced. One study [28] dealt with CD44v6-CAR T cells activated towards target-negative cell line HL-60. The authors managed to reduce the tonic signaling by supplementing the extracellular hinge of CAR with a fragment of low-affinity nerve-growth-factor receptor, and thereby suppress the non-specific killing. Thus, the specificity of CAR T cell-mediated killing can be tremendously enhanced by deliberate combination of original human sequences in chimeric receptor constructs. As our Flt3-CAR construct comprises the non-mutated full-length Flt3Lg sequence, the chimeric receptor is supposed to interact with Flt3 as a fully identical equivalent of Flt3Lg [28].

Leukemic blasts may adapt to immunotherapy through mutations in the target epitope. Considering the outgrowth of AML clones resistant to Flt3-CAR T cell therapy, it is reasonable to expect that such clones will have a defective ligand binding site in the Flt3 molecule. Since Flt3Lg stimulates proliferation in AML (as we demonstrate by using an in vitro model), disruption of the Flt3Lg binding site in Flt3 may deprive blast cells of the proliferative advantage.

Apart from that, we should mention the prognostic significance of the endogenous Flt3Lg levels for AML patients undergoing induction chemotherapy [29]. According to the study, a sustained increase in the concentration of soluble Flt3Lg in the blood plasma (up to 16 ng/mL on day 22 of chemotherapy) correlates with better prognosis. By contrast, unsteady kinetics or consistently low plasma levels of Flt3Lg (within the total range of 0.2–9.5 ng/mL) indicate increased likelihood of refractoriness or relapse. Within the specified range of soluble Flt3Lg concentrations, the Flt3-CAR T cells can efficiently recognize and kill their AML targets.

In summary, our results provide a proof-of-concept that a full-length cytokine sequence within chimeric antigen receptor allows targeting corresponding growth factor receptors with high specificity and affinity. In particular, Flt3-CAR T cells can be used to eliminate AML blast cells with prominent surface expression of Flt3. Indications for such therapy may involve endogenous blood levels of soluble Flt3Lg. AML patients with low blood levels of soluble Flt3Lg (incidentally associated with high risks of relapsed/refractory disease) are more likely to respond. Further research, and notably in vivo testing, will be required to define the clinical prospects of the developed approach.

## Data Availability

The raw data supporting the conclusions of this article are available on request, without undue reservation.
